# Biofilm formation of *Pasteurella multocida* on bentonite clay

**Published:** 2013-06

**Authors:** Ramachandranpillai Rajagopal, Govindapillai Krishnan Nair, Mangattumuruppel Mini, Leo Joseph, Mapranath Raghavan Saseendranath, Koshy John

**Affiliations:** 1 Department of Veterinary Microbiology, College of Veterinary and Animal Sciences, Mannuthy, Thrissur, Kerala, India, 680 651; 2 Kerala Agricultural University Poultry Farm, College of Veterinary and Animal Sciences, Mannuthy, Thrissur, Kerala, India, 680 651; 3 Department of Preventive Medicine and Veterinary Epidemiology, College of Veterinary & Animal Sciences, Mannuthy, Thrissur, Kerala, India. 680 651; 4 Department of Veterinary Microbiology, College of Veterinary and Animal Sciences, Pookot, Lakkidi P.O., Wayanad, Kerala, India - 673 576

**Keywords:** biofilm, *Pasteurella multocida*, bentonite clay, capsular polysaccharide, Maneval staining

## Abstract

**Background and objectives:**

Biofilms are structural communities of bacterial cells enshrined in a self produced polymeric matrix. The studies on biofilm formation of *Pasteurella multocida* have become imperative since it is a respiratory pathogen and its biofilm mode could possibly be one of its virulence factors for survival inside a host. The present study describes a biofilm assay for *P. multocida* on inert hydrophilic material called bentonite clay.

**Materials and methods:**

The potential of the organism to form *in vitro* biofilm was assessed by growing the organism under nutrient restriction along with the inert substrate bentonite clay, which will provide a surface for attachment. For quantification of biofilm, plate count by the spread plate method was employed. Capsule production of the attached bacteria was demonstrated by light microscopic examination following Maneval staining and capsular polysaccharide estimation was done using standard procedures.

**Results and Conclusion:**

The biofilm formation peaked on the third day of incubation (1.54 ×10^6^ cfu/g of bentonite clay) while the planktonic cells were found to be at a maximum on day one post inoculation (8.10 ×10^8^ cfu/ml of the broth). Maneval staining of late logarithmic phase biofilm cultures revealed large aggregates of bacterial cells, bacteria appearing as chains or as a meshwork. The capsular polysaccharide estimation of biofilm cells revealed a 3.25 times increase over the planktonic bacteria. The biofilm cells cultured on solid media also produced some exclusive colony morphotypes.

## INTRODUCTION

Scientists no longer consider bacteria to exist exclusively as free living individual entities. Bacteria can exist as films of sessile conglomerations, aptly called biofilms. Biofilms are microbially derived sessile communities characterized by cells that are irreversibly attached to a substratum or to each other, embedded in a self produced matrix of extracellular polymeric substances (1). The list of biofilm formers is getting populated rapidly following the footsteps of classical biofilm formers like *Pseudomonas, Vibrio, E. coli* and *Salmonella*.

Bacteria in biofilm mode undergo conspicuous changes in their genetic and phenotypic expression by expressing many novel proteins constituted by outer membrane and heat shock proteins (2), which also may be conserved. Biofilms exhibit remarkable properties such as increased resistance to host defenses, biocides, antibiotics and various physicochemical agents (3).

Biofilms could cause chronic and recrudescent infections, hardly controllable by treatment. Biofilm-forming ability has been increasingly recognized as an important virulence factor *in vivo* for many microorganisms, especially *Staphylococcus* (4) and *Pseudomonas aeruginosa* in cystic fibrotic lungs (5), by facilitating their persistence in the host, evading its defenses and allowing bacterial survival at high antimicrobial concentrations. The thick exopolysaccharide covering not only protect the bacteria but even function as a primary adhesin. Several substrates are used for triggering the bacteria to attach *in vitro*, which may be hydrophobic or hydrophilic. Some of them are polystyrene or PVC plates, flow cells, chitin flakes, stainless steel and glass surfaces.

Studies on biofilm formation of *P. multocida* have become imperative since it is a respiratory pathogen and biofilm mode could possibly be one of its virulence factors for survival inside host. The present study was undertaken to quantify biofilm formation of *P. multocida* on bentonite clay and measure subsequent capsular polysaccharide production.

## MATERIALS AND METHODS

The organism used in this study was *Pasteurella multocida* A: 1 strain (DP1) a very virulent strain isolated from Niranam Duck farm (Pathanamthitta District, Kerala State, India), serotyped at IVRI, Izatnagar and maintained in the Department of Veterinary Microbiology, College of Veterinary and Animal Sciences, Mannuthy, Kerala. Purity of the isolate was checked based on morphology, cultural and biochemical characteristics as described by Barrow and Feltham (6).

### Biofilm assay

The biofilm assay was performed as described by Arun and Krishnappa (7) with some modifications. Briefly, Tryptone soya broth (0.32 per cent) (Himedia, India) supplemented with 0.3 per cent bentonite clay powder (Merck, India) was inoculated with overnight cultures of DP 1 and incubated at 42°C for three days in an Orbital Shaker (Thermocon Instruments Private Limited, Bangalore, India), providing occasional shaking for one hour at 50 rpm. The flasks were incubated for one, three and six days separately. For each day, the experiment was done in triplicate. The attached bacterial mass was then harvested by low speed centrifugation (600 × g for 10 min), washed several times in PBS (pH 7.4) and separated from the bentonite clay by vigorous vortexing. The biofilm formation was quantified by plate counting employing spread plate technique.

### Capsule staining of biofilm cells

Capsule or exopolysaccharide production was detected using congo red-acid fuchsin staining procedure (Maneval staining) for acidic polysaccharides (8).

### Extraction of capsular polysaccharide

Extraction procedures were done as per the method employed by Chung *et al*. (9) with some modifications. The planktonic and biofilm organisms were harvested in PBS (pH 7.4) and washed thrice in the same buffer by centrifugation at 10,000 x g for 15 min and finally re-suspended in 2.5 per cent sodium chloride solution to yield 3 × 10^9^ cells/ml. The suspension was then kept at 56°C for one hour in boiling water bath. It was then centrifuged at 17,000 × g for 20 min and the supernatant was collected and dialyzed for 48 h against 0.85 per cent sodium chloride solution containing 0.01 per cent Thiomersal (Merck, India), using a 10 kDa molecular mass cut-off dialysis membrane (Sigma Aldrich, United States) at 4°C. The product was then concentrated to half the volume using poly vinyl pyrollidone (Merck, India) and filtered through a 0.2µ membrane filter (Millipore, United States); the filtrate was designated as crude capsular extract. The extractions were done in triplicate.

### Characterization of crude capsular extract (CCE)

The total carbohydrate content of crude capsular extract of planktonic and biofilm cells was measured by phenol sulphuric acid method using glucose, mannose, ribose and galactose (Merck, India) as standard sugars (10).

## RESULTS

### Biofilm assay

The biofilm cells reached a peak on the third day of incubation with an average count of 1.54 ×10^6^ cfu/g of BC while the planktonic cells were found to be maximum on day one post inoculation, with a peak count averaging about 8.10 ×10^8^ cfu/ml of the broth. The results of the plate count of biofilm and planktonic cells on days 1, 3 and 6 post inoculation are shown in Table 1.

**Table 1:** Plate count for biofilm and planktonic cells of *P. multocida* A:1.

Duration of incubation (Days)	Plate count*	

	Biofilm cells (cfu/g)**	Planktonic cells (cfu/ml)
1	5 .0 ×10^4^	8.10 ×10^8^
3	1.54 ×10^6^	1.36 ×10^7^
6	3.81 ×10^5^	8.27 ×10^5^

* Mean of triplicate experiments for each day of incubation.

** Biofilm count expressed as cfu/g of bentonite clay and planktonic cells as cfu/ml.

**Fig. 1:**
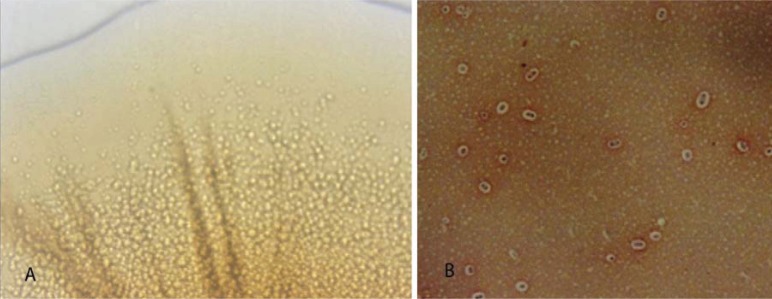
(A) Colony of *P. multocida* biofilm morphotype showing the radiating strands from centre to periphery (40 X magnification). This colony resembles the sectored colony type of the organism growing *in vivo* or that of very virulent strains and was found exclusive to biofilm colonies. (B) Harvested biofilm of *P. multocida* showing very dense and discernible capsule as a white halo around the cells following Maneval Staining.

Some of the biofilm colonies on nutrient agar during plate count consistently showed variation in colony morphology from that of planktonic *P. multocida*. The planktonic cells of *P. multocida* produced circular, convex, smooth and translucent colonies with an entire edge after 24 h at 37°C, while the biofilm colonies were characterized by radiating strands from centre to periphery which were very much discernable when viewed under oblique light (Fig. 1A). The biofilm colonies were smooth and translucent, but the central portion of the colony showed opacity. Some of the biofilm colonies were having an undulating margin, while most of the colonies revealed an entire edge.

### Capsule demonstration

**Fig. 2:**
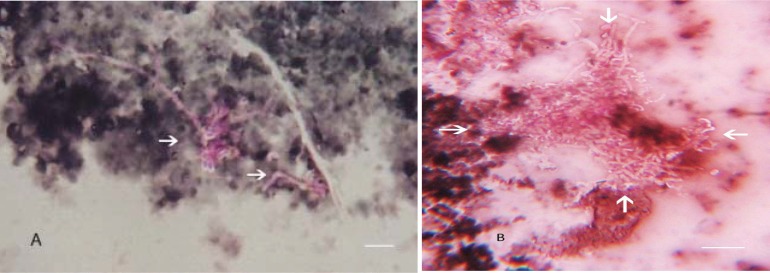
(A) Coccobacillary *P. multocida* organisms with thick dense capsule and appearing as chains attached to bentonite clay (white arrows), Maneval Staining, (100 X). (B) Late biofilm culture revealing organisms that formed a thick mesh work (between the arrows) on bentonite clay (100 X), plausibly due to the formation of exopolysaccharide. Bacteria resist the penetration of stain at this stage.

Maneval staining of late logarithmic phase of three day old biofilm culture of *P. multocida* revealed large aggregates of cocco-bacillary cells. The biofilm cells were heavily capsulated (Fig. 1B), the capsule appeared as a thick refractory halo around the bacterial cells. The staining of late logarithmic phase biofilm cultures evidenced an unusual change in morphology of the bacteria in that they formed chains or a thick meshwork of aggregated cells (Fig. 2A and 2B). The aggregated cells even resisted the penetration of stain so that the bacteria remained unstained in their thick exopolysaccharide covering, which appeared as a dense white halo around the cells.

### Characterization of CCE

The capsular polysaccharide content of biofilm cells was approximately 3.25 times than that of planktonic bacteria grown in TSB, with all the sugar standards (Table 2).

**Table 2:** Carbohydrate content of CCE of *Pasteurella multocida* A:1.

	Concentration of polysaccharides in CCE (µg/ml)*			

	Glucose	Mannose	Ribose	Galactose
Plankton	43.34	36.69	32.20	64.84
Biofilm	140.73	108.76	141.39	154.93

* All the values were obtained by finding the mean of triplicate experiments with each standard sugar

## DISCUSSION

Bacteria will attach to virtually any surface *in vitro*, provided all the other conditions are suitable for attachment. Bentonite clay is an inert material which imbibes water many times its volume forming thick thixotropic gels, thereby providing ample surface area for attachment. Tryptone soya broth is widely recommended as the medium of growth for inducing slime production and biofilm formation (11). Conditions required for biofilm formation like nutrient restriction (12), aeration and uniform mixing (13) were also provided in this study.

The peaking of biofilm cells on the third day of incubation was in accordance with the finding of Vadakel (14). Attachment capabilities of the bacteria was found to be slightly lower when compared to the biofilm count of 1.5 × 10^10^ cfu/g of bentonite clay as described by Vadakel (14). Vadakel (14) also reported a rapid decline of planktonic cells during the subsequent days of incubation, which was not observed in this study. A similar pattern was reported by Anwar *et al*. (15) for *Pseudomonas aeruginosa* in that both biofilm and planktonic population remained constant throughout the study period of seven days. Obviously, *P. multocida* may not be a strong biofilm former like *Pseudomonas* sp., but it could form biofilm *in vitro*. Olson *et al*. (16) opined that for biofilm formation of *P. multocida* in TSB, supplementation with two percent fetal bovine serum (FBS) and 10 percent carbon dioxide tension was required.

Light microscopic studies of biofilm cells following the Maneval staining revealed some morphological differences for the attached cells. Though the chain formation was not reported for *Pasteurella multocida*, it has been reported for other bacteria like *S. enterica* serovar Typhimurium DT 104 biofilm by Rezende *et al*. (17) and similar aggregates of soil bacteria on sand particles by Vandevivere and Kirchman (18). The heavily capsulated aggregated cells even resisted the penetration of stain so that the bacteria remained unstained in their thick exopolysaccharide covering. Prakash *et al*. (19) reported similar refractoriness of *S. gallinarum* biofilm cells to uranyl acetate stain during electron microscopy and a change in architecture from rods to rounded forms.

Many researchers had used Maneval staining method for demonstration of capsule (8, 20, 21). This staining method was comparable to visualization of capsular polysaccharide by electron microscopic assessments of ferritin labeled *P. multocida* cells (22) and for demonstration of capsule by fluorescent antibody technique (FAT) (8).

There are several contrasting reports on whether encapsulation prevents biofilm formation or enhances bacterial attachment. *Vibrio vulnificus* attachment to microtitre plate was enhanced with increased cell surface hydrophobicity (23). Capsulation increases the cell surface hydrophobicity of *P. multocida* as demonstrated by Thies and Champlin (20). This may also be true for DP1, as the biofilm cells revealed extreme capsulation following Maneval staining. The role of exopolysaccharide in biofilm formation is well documented (24). The capsular polysaccharide content of biofilm cells was approximately 3.25 times than that of planktonic bacteria grown in TSB, with all the sugar standards. Considering the fact that EPS and capsule are very difficult to differentiate, the heavy encapsulation of biofilm cells in this study could be extrapolated to the production of exopolysaccharide following biofilm formation. Davey and Duncan (25) proposed that the capsule physically interfered with biofilm formation while Rezende *et al*. (17) were of the opinion that the capsular polysaccharides played an important role in the formation of biofilms.

Under biofilm mode of growth, morphological variations can occur which may even be passed on to the next generation, giving rise to different morphotypes. Such morphotypes were described for several bacterial species but no such literature was available for *P. multocida. Pasteurella multocida* on nutrient agar produced circular, convex, amorphous, greyish yellow and translucent colonies with a smooth, glistening surface and an entire edge (26). Rhoades and Rimler (27) described the different colonial morphology of *P. multocida* isolated from birds with fowl cholera as iridescent, sectored with various intensities of iridescence or blue with little or no iridescence. The biofilm colonies characterized by radiating strands from centre to periphery resembled the sectored colony type of *P. multocida*. The colony morphotypes obtained in biofilm were gradually lost in subsequent subcultures and it could be due to the weaker strength of biofilm formed *in vitro* by DP1.

Bacteria forming biofilms are getting more and more elusive and scientists are now trying to put reins on the diseases caused by them by employing more and more sophisticated strategies like genomic and proteomic analyses. The understanding of the complex repertoire of biofilm formation is of paramount importance and then only suitable strategies could be devised to control bacterial diseases. The present study is only a preliminary investigation which provides some perspective on the biofilm formation of *P. multocida* and warrants elaborate studies on the biofilm formation of this organism.
